# HTLV-1 p12 is cleaved to p8 by the signal peptidase complex and its inhibition impairs p8-dependent transmission

**DOI:** 10.1371/journal.ppat.1013570

**Published:** 2025-10-10

**Authors:** Florian Simon, Norbert Donhauser, Laura M. Kemeter, Franziska Wittdorf, Sebastian Millen, Heinrich Sticht, Andrea K. Thoma-Kress

**Affiliations:** 1 Harald zur Hausen Institute of Virology, Uniklinikum Erlangen, Friedrich-Alexander-Universität Erlangen-Nürnberg (FAU), Erlangen, Germany; 2 Institute of Biochemistry, Friedrich-Alexander-Universität Erlangen-Nürnberg (FAU), Erlangen, Germany; 3 FAU Profile Center Immunomedicine (FAU I-MED), Friedrich-Alexander-Universität Erlangen-Nürnberg (FAU), Erlangen, Germany; Imperial College London Faculty of Medicine, UNITED KINGDOM OF GREAT BRITAIN AND NORTHERN IRELAND

## Abstract

Infection with the oncogenic delta-retrovirus Human T-cell Leukemia Virus Type 1 (HTLV-1) causes aggressive CD4 + T-cell malignancy or progressive neuroinflammatory disorders after a long latency period. The HTLV-1 accessory protein p8 is cleaved from its precursor p12, and both proteins are required for viral persistence. Moreover, p8 enhances viral infectivity by inducing cell-cell contacts and cellular conduit formation. To date, host factors cleaving p12 to p8 remain unknown. Here, we report that p12 carries a signal peptide that is cleaved by the signal peptidase complex (SPC) to generate p8, and blocking of p12 cleavage correlated with a decreased cell aggregation and conduit formation, leading to impaired viral transmission of chronically HTLV-1 infected MT-2 cells. Bioinformatics identified p12 to carry a signal peptide, which is cleaved to generate p8. Inhibition of the SPC function by the SPC-specific inhibitor Cavinafungin and transient knockdown of SPC subunits confirmed the importance of the SPC to cleave p12 to p8. Mutational studies of the signal peptide sequence based on bioinformatics predictions generated cleavage-deficient p12 mutants and verified critical residues for signal peptide cleavage. Further, co-culture assays between Cavinafungin pre-treated chronically infected MT-2 cells, which transmit HTLV-1 dependent on p8, and Jurkat T-cells revealed a significantly impaired viral cell-cell transmission, suggesting that blockage of p12 cleavage interferes with p8-dependent HTLV-1 transmission. Imaging analysis confirmed that SPC-inhibition impairs cell aggregation in MT-2 cells and blocks p8-induced conduit formation in transfected Jurkat T-cells. Collectively, we identified the SPC as the host cell factor cleaving p12 to p8. Inhibition of p12 cleavage led to the absence of p8, which led to impaired cell-to-cell transmission, and coincided with the absence of p8-induced cell aggregation and conduit formation.

## Author summary

The delta-retrovirus Human T-cell Leukemia Virus Type 1 (HTLV-1) encodes the accessory protein p8, which is generated by proteolytic cleavage of its precursor p12. Earlier work has shown that p8 enhances cell-cell contacts and the formation of cellular conduits, thereby increasing HTLV-1 transmission. Our work identified that p12 is cleaved by the signal peptidase complex (SPC) to generate p8. Since the SPC is known to facilitate maturation of secretory and membrane-resident proteins, generation of p8 by proteolytic cleavage of p12 via the SPC is an efficient strategy to exploit the host cell machinery. Given that p8 is important for virus transmission, and SPC-inhibition resulted in an impaired HTLV-1 transmission, we propose the SPC as a new cellular target to counteract HTLV-1 cell-cell transmission. Moreover, we were able to generate cleavage-deficient p12 mutants, which allow to study the functions of p12 and p8 independently of each other.

## Introduction

Human T-cell Leukemia Virus Type 1 (HTLV-1) is an oncogenic retrovirus that primarily targets CD4 + T-cells [1]. An estimated 5–20 million people worldwide are currently infected, and the most prevalent genotype worldwide is HTLV-1a [2]. After an extensive clinical latency period of up to 40 years, about 5% of carriers develop either adult T-cell leukemia/lymphoma (ATLL), an extremely aggressive CD4 + T-cell malignancy, or HTLV-1-associated myelopathy/tropical spastic paraparesis (HAM/TSP), a progressive neuroinflammatory disorder [3–6]. Although HTLV-1 persists asymptomatically in most infected individuals, emerging data indicate higher morbidity and mortality rate in asymptomatic carriers [7]. HTLV-1 is transmitted via cell-containing bodily fluids including blood, semen, and breast milk [8]. While cell-free HTLV-1 transmission is inefficient and free virions are hardly detected in infected individuals [9–11], HTLV-1 requires close cell-to-cell contacts to infect T-cells via virological synapses, viral biofilms or cellular conduits, enabling efficient transmission of HTLV-1a [12–14].

The *open reading frame I* (*ORF-I*) encoded viral proteins p12 and p8 play an essential role in HTLV-1a transmission and persistence in the macaque model, while being dispensable for viral replication *in vitro* [15–18]. *In vivo*, p12 and p8 counteract cytotoxic CD8 and NK cell responses, as well as the clearance of infected cells by monocytes in the host, thereby favoring viral persistence [19,20]. Furthermore, p8 has been shown to increase T-cell adhesiveness and the number of cell-cell contacts, thus, enhancing HTLV-1 transmission via close cell-to-cell contacts [14,21]. In addition, p8 induces the formation of cellular conduits and tunneling nanotubes (TNTs), which are supposed to facilitate the transfer of p8 alongside with HTLV-1 virions to uninfected cells, thereby boosting HTLV-1 transmission via distant cell-to-cell contacts [14,22–24]. Transfer of p8 to uninfected T-cells is proposed to trigger anergy, therefore enhancing susceptibility to HTLV-1 infection [14].

The p12 protein is a highly hydrophobic 99-amino acid (aa) protein that is resident to the endoplasmic reticulum (ER) and cleaved between aa 29 and 30 to generate the 70-aa protein p8, which is localized at the plasma membrane [25]. Importantly, p12 has never been described to be fully cleaved to p8. Instead, T-cells transfected with wildtype *ORF-I* DNA (p12WT) express 60% p12 and 40% p8 protein [25,26]. In infected patients, a dysbalanced p12 to p8 ratio is associated with a lower proviral load [26], which is an important predictor for disease development [27–29]. Furthermore, altering the p12 to p8 ratio by mutation restored the susceptibility of infected cells to CTL responses *in vitro* [26], suggesting that manipulation of p12 cleavage could interfere with the establishment of infection and restore clearance of infected cells by CTL responses. There are several naturally occurring mutants described that impact p12 cleavage and therefore alter the p12 to p8 ratio. For example, the p12G29S mutant shows compromised p12 cleavage and expresses predominantly p12, whereas the p12D26N mutant displays enhanced p12 cleavage and predominantly expresses p8 [26]. While a deletion mutant of the first 29 aa (p12delta29) has been generated to study the functions of p8 [25], mutants exclusively expressing p12 are lacking and impede the study of functions of p12.

In this study, we aimed to identify the cryptic host cell factor cleaving p12 to p8, and we hypothesize that inhibition of p12 cleavage interferes with HTLV-1 cell-to-cell transmission. Here, we show that p12 is a substrate of the signal peptidase complex (SPC), and p12 cleavage inhibition leads to an absence of p8, which in turn decreases p8-dependent HTLV-1 transmission via impaired T-cell adhesiveness and conduit formation. Based on these findings, we generated the first p12 cleavage deficient mutants, allowing to exclusively study the functions of p12 independent of p8. Taken together, uncovering the SPC as the host cell factor cleaving p12 provides the foundation for the development of strategies to interfere with HTLV-1 transmission and the establishment of persistent infections.

## Results

### p12 is cleaved to p8 via the signal peptidase complex (SPC)

N-terminal cleavage of the first 29 aa of the ER resident p12 protein generates the plasma membrane associated p8 protein [25]. To identify the host factors cleaving p12 to p8, we searched for cellular peptidases that recognize the cleavage site of p12 with the peptidase database MEROPS without retrieving any hits. Moreover, use of a commercial protease inhibitor screen including 15 broad spectrum protease inhibitors did not affect cleavage of p12 to p8 (S1 Fig).

**Fig 1:**
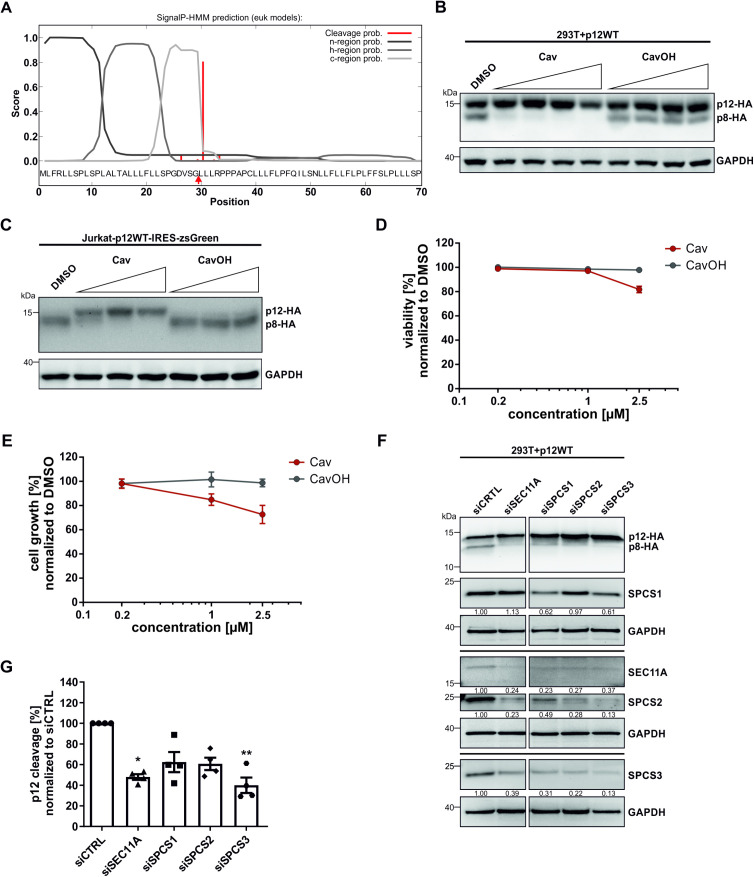
p12 carries a signal peptide that is cleaved via the signal peptidase complex (SPC). **(A)** SignalP hidden Markov model (HMM) output of amino acids (aa) 1-70 is shown. Signal peptide probability: 0.951; Max cleavage site probability: 0.805 between aa 29 and 30. **(B)** Representative immunoblot of p12 cleavage in p12-transfected 293T cells treated with dimethyl sulfoxide (DMSO) or increasing doses (0.05 µM, 0.1 µM, 0.2 µM or 1µM) of either Cavinafungin (Cav) or Cavinafungol (CavOH) for 18 h. **(C-E)** Representative immunoblot of p12 cleavage **(C)**, cell viability **(D)** and growth **(E)** of Jurkat cells stably expressing p12 (Jurkat-p12WT-IRES-zsGreen) treated with DMSO and increasing doses of either Cav or CavOH (0.2 µM, 1 µM or 2.5 µM) for 48 h. Data shown as means ± SEM. n=3. **(F-G)** Representative immunoblot **(F)** and densitometric analysis **(G)** of p12 cleavage in 293T cells transfected with siRNA targeting SPC subunits. Data shown as means ± SEM. n=4. Statistical significance was determined using Kruskal-Wallis test. *p<0.05, **p<0.01.

Since p12 is ER resident [25], we focused on ER-resident proteases capable of cleaving p12. In general, ER translocation of newly translated proteins is facilitated by signal peptides [30]. Therefore, we hypothesized p12 to encode a signal peptide in order to translocate to the ER. To test if p12 indeed carries a signal peptide, computational analysis was performed using the signal peptide prediction server SignalP 3.0 [31,32]. We found that the first 29 aa of p12 were predicted to be a signal peptide with a probability of 95% (Fig 1A). This prediction was confirmed by PrediSi [33], another tool for predicting signal peptides (S2 Fig). The signal peptidase complex (SPC) resides in the ER membrane and is known to cleave off N-terminal signal peptides [34]. Thus, we hypothesized that p12 is cleaved to p8 by the SPC. To confirm this, we transfected HEK-293T cells with p12WT expression cassettes and treated the cells with the specific SPC-inhibitor Cavinafungin (Cav) or its inactive derivate Cavinafungol (CavOH) (Fig 1B). Cav is a fungal lipopeptide isolated from *Colispora cavincola*, which specifically binds to the SPC catalytic subunit and thereby inhibits SPC function [35,36]. CavOH is a reduced derivate of Cav which cannot bind to the SPC catalytic subunit and serves as technical control [35]. In line with our hypothesis, p12 to p8 processing was strongly inhibited by Cav but not by CavOH, suggesting that p12 is indeed carrying a signal peptide that is cleaved via the SPC to generate p8 (Fig 1B). Since CD4 + T-cells are the main target cell population of HTLV-1 *in vivo* [1], we aimed to verify the previous finding in the CD4 + Jurkat T-cell line and transduced the cells to stably express C-terminally HA-tagged p12. Interestingly, in these cells, p12 was fully cleaved to p8 (Fig 1C, DMSO), suggesting that cleavage of p12 is either faster or more efficient in Jurkat T-cells compared to other cell types. Nevertheless, treatment with Cav but not CavOH dose-dependently recovered p12 expression and inhibited p12 cleavage (Fig 1C). In addition, treatment of Jurkat T-cells with up to 1 µM Cav had no effect on cell viability (Fig 1D), but reduced cell growth by 20% (Fig 1E). Up to 2.5 µM Cav decreased cell viability by 20% and reduced cell growth by 30% (Fig 1E).

To confirm that p12 is cleaved by the SPC independent of a compound, we interfered with SPC subunit expression via small interfering RNA (siRNA). The SPC comprises five subunits: the accessory subunits SPC12 (SPCS1), SPC25 (SPCS2), SPC22/23 (SPCS3) and catalytic subunits SEC11A and SEC11C [37]. Further, the SPC exists in two paralogous complexes with both complexes consisting of the accessory subunits but only either catalytic SEC11A (SPC-A) or SEC11C (SPC-C) [38]. We transfected HEK-293T cells with siRNAs targeting each subunit separately before transfecting the cells with p12WT expression plasmids. Respective immunoblots analyzed protein expression of p12 and SPC subunits (Fig 1F). Transient knockdown reduced SPC subunit expression to varying degrees, ranging from 0.62-fold expression of SPCS1 to 0.13-fold expression of SPCS3. Expression of SEC11C was not affected, indicating that the used siRNA pool did not downregulate SEC11C (S3 Fig). Downregulation of any SPC subunit did also affect protein expression of other subunits, e.g., SPCS3 knockdown led to downregulation of SPCS1, SPCS2 and SEC11A, suggesting that knockdown of singular subunits might interfere with SPC stability (Fig 1F). Densitometric analysis confirmed that downregulation of either regulatory subunits SPCS1, SPCS2, or SPCS3 or catalytic subunit SEC11A correlated with a significantly impaired p12 cleavage (Fig 1G). Together, bioinformatic predictions, a SPC-specific compound and silencing of SPC subunits indicate that p12 is cleaved by the SPC.

### Two residues, V27 and G29, are critical for p12 cleavage

**Fig 2:**
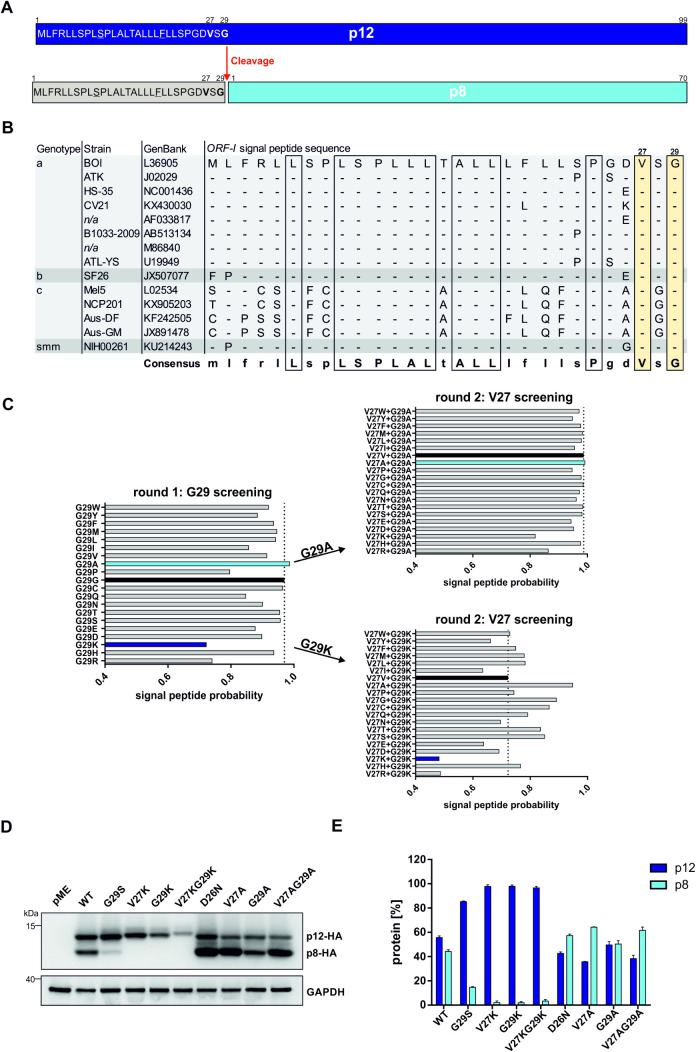
Mutation of conserved V27 or G29 abrogates cleavage of p12. **(A)** Signal peptide sequence of p12. **(B)** Amino acid sequence alignment of the *ORF-I* signal peptide sequence of various HTLV-1 strains and genotypes. *n/a*: not available. **(C)** *In silico* screening for amino acids showing highest or lowest signal peptide probability at position 27 and 29 using SignalP 3.0. **(D, E)** Representative immunoblot **(D)** and densitometric analysis **(E)** of p12 cleavage in generated mutants in 293T cells. Data shown as means ± SEM. n = 3.

The main function of the SPC is to remove SPs from proteins entering the ER [34]. Generally, the sequences of SPs vary in composition and length. Therefore, SPs are defined by three characteristic regions: (i) an often positively charged n-region at the N-terminus, (ii) a 7–15 aa hydrophobic h-region and (iii) a polar c-region containing the cleavage site [39]. Importantly, previous studies on the SPC and signal peptides indicate that aa -1 and -3 relative to the cleavage site are crucial for cleavage and have to be occupied by small and hydrophobic aa [40,41]. In the p12 protein derived of the HTLV-1a strain BOI, the residues -1 and -3 relative to the scissile bond correspond to V27 and G29 (Fig 2A). We performed sequence alignment analysis of those sequences corresponding to the *ORF-I* signal peptide sequence of HTLV-1a analyzing different strains of the HTLV-1 genotypes a, b and c, as well as the closely to Simian T-cell Leukemia Virus Type 1 (STLV-1) from sooty mangabey (smm) related HTLV-1smm (Fig 2B). Importantly, we found V27 and G29 to be conserved in all sequences analyzed (Fig 2B), and hydrophobicity was comparable among the genotypes (S4 Fig). Of note, while the start codon of *ORF-I* in HTLV-1b and HTLV-1c is mutated, HTLV-1c *ORF-I* is proposed to be expressed via a bicistronic mRNA [42,43].

Based on these findings, we hypothesized to be able to manipulate p12 cleavage by mutation of V27 and G29. With the help of SignalP 3.0, a tool to predict signal peptide cleavage, we aimed to find V27 and G29 point mutants of *ORF-I* which are predicted with the lowest possible signal peptide probability, hypothesizing that those mutants would not be cleaved and therefore exclusively express p12. Vice versa, we also searched for *in silico* mutants being predicted with the highest possible signal peptide probability, hypothesizing that those mutants might be fully cleaved and therefore exclusively express p8. To identify putative mutants with low SP probability, we first screened residue G29 via SignalP 3.0 [31,32]. Here, G29K was predicted to have the lowest SP probability (Fig 2C, left panel, dark blue bar). To discover mutants with even lower SP probability, we introduced the G29K mutation and screened residue V27 in a second round of computational mutagenesis. We found V27KG29K to be predicted with even lower SP probability (Fig 2C, lower right panel, dark blue bar). Likewise, we searched for mutants with the highest SP probability and found G29A and V27AG29A to be predicted with the highest SP probabilities (Fig 2C, light blue bars).

Next, we generated expression constructs for p12 carrying the predicted mutations: p12V27K, p12G29K, p12V27KG29K, p12V27A, p12G29A or p12V27AG29A. We transfected HEK-293T cells with the mutants and analyzed p12 cleavage via immunoblot (Fig 2D). As controls, we included expression vectors of p12WT, as well as of the cleavage-compromised p12G29S mutant and the cleavage-enhanced p12D26N mutant which were described earlier [26]. Densitometric analysis indicated that upon expression of p12WT, p12 was cleaved to 40% into p8 (Fig 2E). Upon expression of p12G29S, p12 was only cleaved to 20%. Strikingly, in p12V27K, p12G29K and p12V27KG29K mutants, p12 cleavage was completely inhibited, suggesting that the predicted signal peptide probability indeed correlates with p12 cleavage. Moreover, this finding was unexpected since p12 mutants that are completely cleavage-deficient have not been described so far. Next, the p12D26N mutant displayed an increased p12 cleavage to 60% p8. In line with our hypothesis, the p12V27A, p12G29A and p12V27AG29A mutants showed enhanced p12 cleavage, confirming signal peptide prediction via SignalP 3.0 as tool for prediction of p12 cleavage. Taken together, these findings indicate that manipulation of the signal peptide by mutation of conserved residues V27 and G29 alters p12 cleavage, thereby confirming that p12 indeed carries a signal peptide that is cleaved by the SPC. Furthermore, the p12V27K, p12G29K and p12V27KG29K mutants are the first ever-described mutants that display no p12 cleavage and exclusively express p12, which will allow to study the functions of p12 independent of p8 in future experiments.

### p12 cleavage inhibition impairs p8-dependent HTLV-1 cell-to-cell transmission

**Fig 3:**
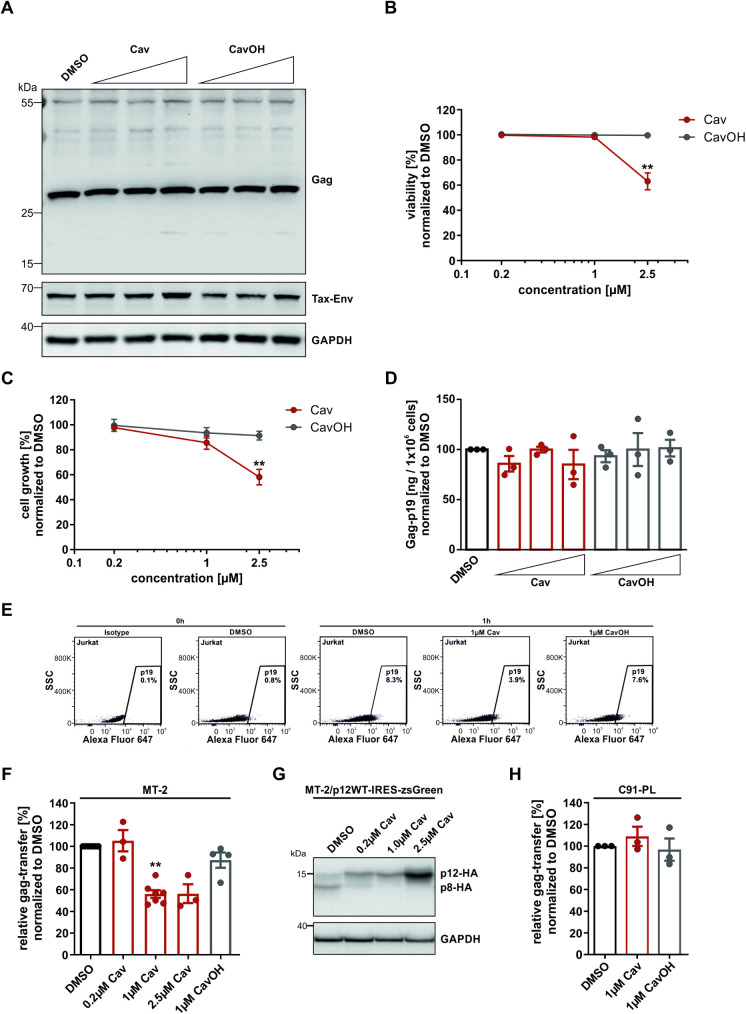
Inhibition of p12 cleavage impairs HTLV-1 cell-to-cell transmission of chronically infected MT-2, but not C91-PL cells. **(A-D)** Viral protein expression **(A)**, cell viability **(B)** and growth **(C)**, as well as virus release **(D)** of chronically HTLV-1 infected MT-2 cells treated with DMSO or increasing doses (0.2 µM, 1 µM or 2.5 µM) of either Cav or CavOH after 48 h of treatment. **(B-D)** Data shown as means ± SEM. n = 3. Statistical significance was determined using Kruskal-Wallis test. *p < 0.05. **(E-F)** Representative scatterplots of gag-positive Jurkat T-cells **(E)** and quantitative analysis **(F)** of virus cell-cell-transmission between Cav or CavOH pretreated MT-2 and Jurkat cells. Data shown as means ± SEM. n ≥ 3. Statistical significance was determined using Kruskal-Wallis test. *p < 0.05, **p < 0.01. **(G)** Representative immunoblot of p12 cleavage in MT-2 cells stably expressing p12 (MT-2-p12WT-IRES-zsGreen) treated with DMSO and increasing doses of Cav (0.2 µM, 1 µM or 2.5 µM) for 24 **h. (H)** Quantitative analysis of virus cell-cell-transmission between Cav or CavOH pretreated C91-PL and Jurkat cells. Data shown as means ± SEM. n = 3.

To analyze the effects of SPC-inhibition in chronically HTLV-1 infected T-cells, we aimed to inhibit p12 cleavage in MT-2 cells. Importantly, MT-2 cells carry a provirus with the p12D26N mutation, which preferentially expresses p8 [23]. In addition, MT-2 cells were reported to form more tunneling nanotube (TNT)-like protrusive structures than other HTLV-1 infected cell lines, and TNT inhibition impaired HTLV-1 transmission [23]. Thus, MT-2 cells were treated with increasing doses of Cav or CavOH for 48 h. Immunoblot analysis indicated no effects on the expression of the viral proteins Gag p55 or Tax-Env (Fig 3A). In addition, processing of the structural Gag p55 polyprotein to p27 or p19 was unaffected (Fig 3A). Furthermore, low concentrations of Cav (up to 1 µM) did not show any effect on cell viability (Fig 3B) and cell growth (Fig 3C), while at higher concentrations of Cav (2.5 µM), both cell viability and cell growth significantly declined (Fig 3B and 3C). Cav treatment did not affect virus release measured via Gag-p19 ELISA at 48h post treatment (Fig 3D). Importantly, when analyzing HTLV-1 cell-to-cell transmission by co-culturing 1 µM Cav-pretreated MT-2 cells with Jurkat T-cells, Gag-transfer was strongly reduced to 55% (Figs 3E, 3F and S5 Fig), suggesting that SPC inhibition impairs virus transmission, potentially by inhibiting p12 cleavage in chronically infected cells. Of note, pretreatment of MT-2 cells with 0.2 µM Cav had no effect on viral transmission while pretreatment with 2.5 µM Cav led to a reduction of cell-to-cell transmission comparable to 1 µM Cav (Fig 3F). Subsequent analysis of p12 cleavage in MT-2 transduced to stably express p12-HA indicated that 0.2 µM Cav does not fully block cleavage, suggesting that full cleavage inhibition of p12 resulting in a complete absence of p8 is needed to impair viral transmission (Fig 3G). However, besides p12, the HTLV-1 envelope precursor protein gp62 is also encoding a signal peptide [44]. Therefore, to exclude unspecific effects of Cav, e.g., by impairing Env processing, we analyzed HTLV-1 cell-to-cell transmission in chronically infected C91-PL cells that are known to transmit HTLV-1 *in vitro* predominantly via viral biofilms [13,45–47], and therefore independently of p8. Treatment of C91-PL with up to 1 µM Cav had no effect on protein expression of viral Gag and Tax, cell viability and cell growth, or viral Gag-p19 release (S6 Fig). In co-culture experiments between C91-PL and Jurkat T-cells, Cav pre-treatment did not affect cell-to-cell transmission, suggesting that short-term SPC-inhibition per se does not interfere with HTLV-1 cell-to-cell transmission (Fig 3H). In conclusion, our data indicate that short-term SPC-inhibition with 1 µM Cav has neither effect on HTLV-1 protein expression and viral release, nor on cell survival and growth. Importantly, Cav-treatment led to significantly impaired viral cell-to-cell transmission in MT-2 cells known to transmit HTLV-1 in a p8-dependent manner [23], while cell-to-cell transmission in C91-PL cells that transmit HTLV-1 independent of p8, was unaffected. Collectively, this suggests that SPC-inhibition interferes with p8-dependent HTLV-1 transmission of MT-2 cells by blocking p12 cleavage, while p8-independent transmission of C91-PL cells is unaffected.

### p12 cleavage inhibition abolishes p8-induced conduit formation and cell aggregation

**Fig 4:**
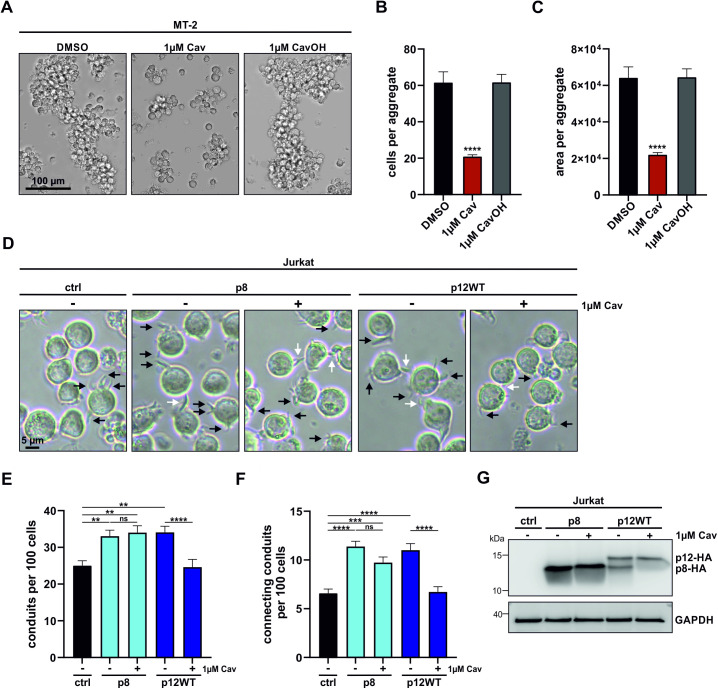
Inhibition of p12 cleavage abolishes p8-induced conduit formation and cell aggregation. **(A-C)** Representative images **(A)** of MT-2 cells treated with DMSO, Cav or CavOH. Cell count **(B)** and area **(C)** per aggregate were quantified. Data shown as means ± SEM of at least 35 images. Statistical significance determined was using Kruskal-Wallis test. *p < 0.05, **p < 0.01, ***p < 0.001, ****p < 0.0001. **(D-G)** Representative images **(D)** of conduit forming Jurkat T-cells transfected with control, p8 or p12WT, and treated with DMSO or Cav. Black arrows indicate conduits, and white arrows indicate connecting conduits. Total conduits **(E)** and connecting conduits **(F)** were quantified. Data shown as means ± SEM of at least 44 images. Statistical significance was determined using Kruskal-Wallis test. *p < 0.05, **p < 0.01, ***p < 0.001, ****p < 0.0001. **(G)** Representative immunoblot confirming p12 cleavage inhibition upon Cav treatment.

The p8 protein is enhancing viral transmission via increasing T-cell aggregation and inducing conduit formation [14,23,24]. Therefore, we hypothesized that SPC inhibition decreases viral cell-to-cell transmission by interfering with cell aggregation and conduit formation. First, we tested the impact of SPC inhibition on the morphology of chronically infected MT-2. Cells were treated with Cav, and unfixed cells were analyzed via transmitted light microscopy (Fig 4A). As reported previously, MT-2 cells are heavily interconnected by conduits [23], which leads to the formation of large cell aggregates. Treatment with Cav, but not CavOH, significantly reduced aggregate size, both by absolute cell number (Fig 4B) as well as aggregate area (Fig 4C), suggesting that SPC-inhibition leads to an absence of p8-induced cell aggregation. Next, we investigated whether a block in p12 cleavage indeed interferes with p8-induced conduit formation. However, due to strong cell aggregation, we were not able to investigate this in MT-2 cells. Therefore, we transfected Jurkat T-cells with p12WT expression plasmids and treated the cells with Cav or solvent control DMSO. Besides empty vector control, we included p8-transfected cells and treated the cells with Cav, as well. Unfixed cells were analyzed via transmitted light microscopy (Fig 4D). Conduits were marked with black arrows, and connecting conduits between two cells were indicated with white arrows (Fig 4D). In line with previous reports [14,24], we found expression of p8 to enhance both the total number of conduits per cell (Fig 4E) as well as the number of conduits interconnecting two cells (Fig 4F). Cav treatment of p8-expressing cells did not affect the number of total conduits (Fig 4E), or connecting conduits (Fig 4F), suggesting that Cav treatment alone has no effect on conduit formation. Expression of p12, which is partly cleaved to p8, did significantly increase both the number of total conduits (Fig 4E), as well as interconnecting conduits (Fig 4F). Importantly, Cav treatment of p12WT-transfected cells decreased the number of both total (Fig 4E) and connecting conduits (Fig 4F) back to the level of empty vector control, likely due to the absence of p8, which was verified via immunoblot analysis (Fig 4G). To conclude, inhibition of p12 cleavage leads to absence of p8, which coincides with impaired cell aggregate formation in chronically infected MT-2 cells and loss of p8-induced conduit formation in Jurkat T-cells.

## Discussion

HTLV-1 establishes persistent infections leading to severe and incurable diseases in up to 10% of carriers [7]. Despite their low expression levels, the accessory proteins p12 and p8 both play a critical role in HTLV-1a infection *in vivo*. Here, p12 and p8 exert a plethora of functions [48], which lead to the establishment of persistent infections [17,20,26]. Moreover, p8 per se enhances viral transmission by increasing cell-to-cell contacts and inducing cellular conduit formation, facilitating transfer of p8 and HTLV-1a virions to uninfected cells [14,22–24]. In addition, transferred p8 is suggested to dampen T-cell responses, thereby possibly priming target cells for infection [14,48,49]. Importantly, without p12 and p8, HTLV-1a infection is non-persistent in the macaque model, which is a relevant animal model system for HTLV-1 infection of humans [17,26], and in infected patients, a dysbalanced p12 to p8 ratio is associated with lower viral burden [26]. Despite their importance in HTLV-1a infection, it was unclear which host cell factors are responsible for cleavage of p12 to p8.

In this study, we identified the SPC to facilitate cleavage of p12 to p8, which is important for HTLV-1 cell-to-cell transmission. Starting from bioinformatics, we identified the first 29 aa of p12 to be a signal peptide (Fig 1A), which is cleaved by the SPC. The SPC is a serine protease that is resistant to standard protease inhibitors [38]. Indeed, we were unable to interfere with p12 cleavage using a commercial protease inhibitor screening (S1 Fig). Only when using the SPC specific inhibitor Cavinafungin (Cav) [35], our data show that p12 cleavage is completely blocked in multiple cell types (Fig 1B and 1C). Subsequently, we validated the importance of the SPC for p12 cleavage by transient knockdown of SPC subunits (Fig 1F and 1G). Despite the SPC comprising two paralogous complexes, SPC-A and SPC-C [38], knockdown of the catalytic subunit SEC11A alone significantly impaired p12 cleavage (Fig 1). In comparable studies investigating SPC substrates, SEC11A knockdown alone did not have an effect on cleavage, and only knockdown of both catalytic subunits SEC11A and SEC11C simultaneously blocked cleavage [50,51]. Our results suggest that SPC-A and SPC-C at least differ in their capacity to cleave p12, which seems to be cleaved via the SPC-A.

In line with our finding that HTLV-1 exploits the SPC to process the accessory protein p12, the HTLV-1 encoded envelope precursor protein gp62 has been previously described to encode a SP [44]. Moreover, several other viruses were reported to exploit the SPC to facilitate maturation of viral polyproteins. In the HTLV-1 related Human Immunodeficiency Virus 1 (HIV-1), the envelope precursor protein gp160 contains a SP, which is cleaved by the SPC to generate gp120 [52]. In the Flaviviridae family, the SPC is required for processing of structural proteins and particle secretion [53]. Here, treatment with Cav potently inhibited Zika and Dengue Virus infection in multiple cell types *in vitro* [35].

Therefore, interfering with SPC function might be a promising antiviral strategy. However, the SPC is an essential protease complex and is central for the biogenesis of secretory and transmembrane proteins, which affects approximately half of the human proteome [54]. SPC-inhibition is expected to interfere with maturation and secretion of key host proteins, including antibodies and hormones [35]. In several model organisms including *Drosophila* and yeast, loss of SPC function was lethal [55–57] and in mammals, the SPC has not been therapeutically targeted yet.

Since p8 has been shown to promote viral transmission [14], we hypothesized that blocking the cleavage of p12 to p8 would interfere with viral transmission. Indeed, we found that SPC-inhibition impaired HTLV-1 cell-to-cell transmission of chronically infected MT-2 cells (Fig 3), which have shown to transmit HTLV-1 dependent on p8 [23]. In subsequent assays, SPC-inhibition coincided with absence of p8-exerted functions including impaired cell adhesion and conduit formation (Fig 4), suggesting that absence of p8 is causative for the impairment of HTLV-1 cell-to-cell transmission. Due to general low expression levels of accessory viral proteins in chronically infected cells [58] and due to absence of p12- and p8-specific antibodies, we could not verify this by direct detection of p12 and p8. Contrary, Cav-treatment did not impair cell-to-cell transmission of C91-PL cells, which transmit the virus predominantly via biofilm-like structures [13, 45-47], and therefore independent of p8. *In vivo*, it is unclear, whether HTLV-1 infects target cells preferentially via cellular conduits, viral biofilms or viral synapses and how the mechanism of transmission is regulated. While our data suggest that SPC-inhibition is able to interfere with p8-dependent cell-to-cell transmission *in vitro*, future work is needed to validate whether absence of p8 leads to decreased viral transmission *in vivo*. Given the relevance of p12 and p8 in immune evasion and viral persistence [19,20,26], future experiments need to address whether blocking p12 cleavage might restore immune recognition and clearance of infected cells.

Moreover, we identified the *ORF-I* signal peptide sequence to be highly conserved among HTLV-1a, b and smm. In the related HTLV-1c genotype, which is prevalent in Central Australia [59], *ORF-I* has no start codon and is proposed to express its gene products via a doubly spliced transcript that incorporates an intact translation start codon in exon 1 of *rex* mRNA to create p16, an alternative protein to p12 [42,48]. Nevertheless, V27 and G29 are present in all HTLV-1 genotypes and strains analyzed, suggesting that cleavage of p12 and related *ORF-I* encoded proteins by the SPC might be conserved. Thus, interfering with p12 cleavage, potentially by SPC-inhibition, might be a promising strategy to limit transmission of other HTLV-1 genotypes as well.

Our computational analysis indicated that the first 29 amino acids cleaved from p12 to generate p8 are a SP (Fig 1). Indeed, we were able to manipulate cleavage of p12 by mutating the SP sequence at conserved positions V27 and G29, which are important for proper cleavage (Fig 2). Although generated mutants p12V27A, p12G29A and p12V27AG29A display increased cleavage of p12 and therefore more p8, p12 mutants that are fully cleaved to p8 are still lacking. Studies investigating p8 so far used a deletion mutant of the first 29 amino acids, p12delta29 [14,22–26,60]. Here, p12delta29 was reported to localize in the cytosol and at the plasma membrane [25]. It was surprising for a hydrophobic protein like p8 [24] to be detected in the hydrophilic cytosol. Now, having discovered that the deleted sequence is a SP sequence, this could explain the cytosolic localization, because the SP is required for proper ER translocation [58]. Eventually, hydrophobic proteins such as p8 are still post-translationally translocated into the ER to prevent aggregation in the cytosol [61]. Although SPs are poorly understood, they may be central to proper protein biogenesis [61]. In fact, SPs are proposed to encode information about the choice of targeting pathway, efficiency of translocation, timing of cleavage, as well as post-cleavage functions [30,61]. Since there are no fully cleaved p12 mutants and p8 can only be analyzed with the SP-deleted mutant p12delta29, some functions of p8 have yet to be discovered.

Importantly, the novel mutants p12V27K, p12G29K and p12V27KG29K were fully cleavage deficient (Fig 2D and 2E). To date, there has not been any p12 mutant described that is not cleaved and therefore exclusively expressing p12. So far, the closest mutant to an exclusive p12 expression was p12G29S mutant that is still cleaved by approximately 20% (Fig 2E). Consequently, all studies about the functions of p12 have used either the p12WT or p12G29S mutant meaning that p8 was still present albeit at low expression levels [23,26,49,62–66]. Discovery of the fully cleavage deficient mutants p12V27K, p12G29K and p12V27KG29K now enables to exclusively study the functions of p12 independent of p8 *in vitro*. To rule out the influence of Cav on other SPC-dependent viral or host proteins, a novel HTLV-1 provirus carrying the mutations in the p12 cleavage site could serve as an alternative to study the transmission of viruses lacking p8 *in vitro* and *in vivo* independently of SPC-inhibition. Beyond, p8-deficient HTLV-1 proviral clones might be used to compare the effects of SPC inhibition on viral transmission between the wild type and mutant proviral clones. Moreover, p8-deficient HTLV-1 proviral clones would allow to investigate the establishment of HTLV-1 persistence in absence of p8 *in vivo*. Based on earlier findings, we would expect absence of p8 to interfere with the establishment of persistent HTLV-1 infections by restoring susceptibility to cytotoxic CD8 + T-cell and NK cell responses in infected cells, consequently contributing to clearance of infected cells [19].

In summary, our research provides evidence that p12 is cleaved via the SPC and inhibition of p12 cleavage leads to a decreased cell-to-cell transmission, which is consistent with the absence of p8-induced cell aggregation and conduit formation. Therefore, targeting p12 processing could represent a promising therapeutic approach, potentially reducing the viral burden in HTLV-1 infected patients.

## Materials and methods

### Cell lines

HEK 293T cells were grown in Dulbecco’s modified eagles medium (Gibco, Life Technologies, Darmstadt, Germany). Jurkat T-cells were cultivated in a 1:1 mixture of Panserin 401 (PAN Biotech GmbH, Aidenbach, Germany) and RPMI-1640 medium (Gibco, Life Technologies). Chronically HTLV-1 infected MT-2 and C91-PL cells were grown in RPMI-1640 medium. All cell culture media were supplemented with 10% fetal calf serum (FCS; Anprotec, Bruckberg, Germany), 1% GlutaMAX, 0.12 mg/ml penicillin and 0.12 mg/ml streptomycin (all Gibco, Life Technologies). Cell lines were tested for mycoplasma contamination. Authenticity of cell lines was verified by short tandem repeat analysis (DSMZ, Braunschweig, Germany). Jurkat T-cells (corresponding to DSMZ ACC 282), 293T cells (DSMZ ACC 635), and MT-2 cells (JCRB1210) have been used before and were kindly provided by Ralph Grassmann (deceased, FAU, Erlangen, Germany) [24], and C91-PL (Cellosaurus CVCL_8342) were kindly provided by Maria-Luisa Calabrò (University of Padova, Padua, Italy).

### Plasmids

pME, pME-p12delta29-HA (pME-p8), pME-p12G29S-HA (pME-p12G29S) and pME-p12WT-HA (pME-p12WT) were kindly provided by Genoveffa Franchini and have been described earlier [17,25,49,60]. psPAX2 (Addgene plasmid #12260) and pMD2.G encoding vesicular stomatitis virus glyocoprotein G (VSV-G; Addgene plasmid #12259) were provided by Didier Trono. pLVX-EF1a-IRES-zsGreen (Clontech, #631982) was purchased from TaKaRa Bio USA. To generate pLVX-p12WT-EF1a-IRES-zsGreen, p12WT sequence was amplified from pME-p12WT-HA including a Kozak Sequence, the C-terminal HA tag and flanking *EcoR*I and *BamH*I restriction sites. The following oligonucleotides were used: EcoRI-p12WT-fwd ATTAGAATTCGCCACCATGCTGTTTCGCCT and zsGreen-BamHI-Stop-p12WT-rev ATATGGATCCTTAGAAGAGGAAAGCCGCGG. The backbone vector was linearized via *EcoR*I and *BamH*I digestion. The oligonucleotides were inserted into pLVX-EF1a-IRES-zsGreen by standard cloning procedures.

pME-p12D26N-HA was generated using site directed mutagenesis on pME-p12WT-HA and oligonucleotides p12D26N-fwd GGCCGCTGACGTTGCCCGGAGAAAG and p12D26N-rev CTTTCTCCGGGCAACGTCAGCGGCC. pME-p12V27K-HA, pME-p12G29K-HA, pME-p12V27KG29K-HA, pME-p12V27A-HA, pME-p12G29A-HA and pME-p12V27AG29A-HA were generated using site directed mutagenesis on pME-p12WT-HA and synthesized by ShineGene Bio-Technologies (Shanghai, China).

### Bioinformatics

MEROPS (https://www.ebi.ac.uk/merops/cgi-bin/specsearch.pl; last access on: January 10, 2025) was used for analyzing the cleavage site of p12 using the p12 sequence depicted in Fig 1A. SignalP 3.0 (https://services.healthtech.dtu.dk/services/SignalP-3.0/; last access on: January 10, 2025) and PrediSi (http://www.predisi.de/; last access on: January 14, 2025) were used for signal peptide prediction [31–33]. For sequence alignment, p12 signal peptide sequences and corresponding sequences were translated of HTLV-1a (GenBank: L36905, J02029, NC001436, KX430030, AF033817, AB513134, M86840, U19949), b (GenBank: JX507077), c (GenBank: L02534, KX905203, KF242505, JX891478) and smm (GenBank: KU214243) genotypes, and sequences were aligned using the Simple Consensus Maker (https://www.hiv.lanl.gov/content/sequence/CONSENSUS/SimpCon.html; last access on: August 13, 2025). Frequency of hydrophobic amino acids within the *ORF-I* signal peptide sequence was analyzed using the Peptide 2.0 Hydrophobicity/Hydrophilicity Analysis tool (https://www.peptide2.com/N_peptide_hydrophobicity_hydrophilicity.php; last access on: August 15, 2025), and grand average of hydropathy (GRAVY) was calculated using the GRAVY calculator (https://www.gravy-calculator.de/index.php; last access on: August 15, 2025).

### DNA transfection

293T cells were seeded at 5^×^10^5^ cells per six-well plate. The next day, the cells were transfected with 2 µg of pME-p12WT using GeneJuice transfection reagent (Merck Millipore, Darmstadt, Germany) according to the manufacturer’s protocol.

1^×^10^7^ Jurkat T-cells were transfected by electroporation with 100 μg DNA using the *Gene Pulser Xcell Electroporation System* (BioRad, München, Germany) at 290 V and 1500 μF with exponential pulse protocol as described previously [24].

### Transduction

293T cells were co-transfected with 3 µg pMD2.G, 6 µg psPAX and 6 µg pLVX-EF1a-p12WT-IRES-zsGreen or empty vector pLVX-EF1a-IRES-zsGreen. After 72 h, lentivirus-containing supernatant was collected and concentrated. 1^×^10^6^ Jurkat T-cells or MT-2 cells were spin-infected with the supernatant for 2 h and cultivated for at least 2 weeks prior to experiments. Jurkat-p12WT or MT-2-p12WT cells were seeded at 5^×^10^6^ cells per six-well plate and treated with Cavinafungin (Cav) or Cavinafungol (CavOH) [35], both dissolved in dimethyl sulfoxide (DMSO).

### Western blotting

Cells were lysed in TNE buffer (10 mM NaCl, 10 mM Tris/HCl (pH 7.0), 10 mM EDTA, 1% Triton X-100, 2 mM DTT) supplemented with protease inhibitors (20 µg/mL leupeptin, 20 µg/mL aprotinin and 1 mM phenylmethylsulfonyl fluoride). Jurkat and 293T cells were subjected to two freeze-thaw cycles in liquid nitrogen. MT-2 and C91-PL cells were additionally sonicated three times for 30 s using Branson Ultrasonics Analog Sonifier Modell 450 (Emerson Electric Co., St. Louis, MO, USA; output control = 8, duty cycle = 60, 3 × 30 s). Cell lysates were centrifuged, and total protein was obtained. Protein concentration was determined by Bradford assay using the Roti-Quant reaction agent (Carl Roth GmbH + Co. KG, Karlsruhe, Germany). 50 µg of protein lysate was loaded onto 12–15% polyacrylamide gels, separated via SDS-PAGE and transferred to a nitrocellulose membrane. Membranes were blocked and incubated with the indicated primary and secondary antibodies. For detection of p12 and p8, HRP-conjugated HA antibody (12013819001; Roche, Mannheim, Germany) was used. Primary antibodies used: anti-GAPDH (sc-47724; Santa Cruz Biotechnology, Dallas, TX, USA), anti-SPCS1 (11847–1-AP; Proteintech, Planegg-Martinsried, Germany), anti-SEC11A (14753–1-AP; Proteintech), anti-SPCS2 (14872–1-AP; Proteintech), anti-SPCS3 (SAB1302993; Sigma-Aldrich, Taufkirchen, Germany), anti-SEC11C (NBP1–80774; Novus biologicals, Wiesbaden, Germany), anti-Gag (0801082; ZeptoMetrix, Buffalo, NY, USA), and anti-Tax-1 (ab26997; Abcam, Cambridge, UK). Secondary antibodies used: HRP-conjugated goat anti-mouse IgG (Amersham, NA9310; GE Healthcare, Chicago, IL, USA), and HRP-conjugated goat anti-rabbit IgG (NA9340; GE Healthcare). Blots were analyzed with in-house ECL solution and imaged with the INTAS Advanced Fluorescence and ECL Imager (INTAS Science Imaging GmbH, Göttingen, Germany). Densitometric analysis was conducted using AIDA Image Analyzer v4.23 (Raytest Isotopenmessgeräte GmbH, Straubenhardt, Germany).

### siRNA transfection

293T cells were seeded at 5^×^10^5^ cells per six well plate. After 24 h and 48 h, cells were transfected with 2 µg siRNA using Lipofectamin2000 (Thermo Fisher Scientific, Carlsbad, CA, USA) according to the manufacturer’s protocol. ON-TARGETplus SMARTpool human siRNA (Dharmacon, Lafayette, CO, USA) for SEC11A (#L-006038-00-0020), SEC11C (#L-005932-00-0020), SPCS1 (#L-020577-00-0020), SPCS2 (#L-020897-00-0020) or SPCS3 (#L-010124-00-0020) and non-targeting control (#D-001810-10-20) was used. After 72 h, cells were transfected with 2 µg pME-p12WT using GeneJuice according to the manufacturer’s protocol. After 96 h, cells were lysed.

### ELISA

Supernatant was collected and sterile filtered (0.45 µm). The HTLV-1/2 p19 Antigen ELISA Kit (ZeptoMetrix, Buffalo, NY, USA) was used according to the manufacturer’s protocol. Samples were analyzed in technical duplicates in at least three independent experiments.

### Co-culture assay

Chronically infected MT-2 or C91-PL cells were treated with 1 µM Cav, CavOH [35] or DMSO for 24 h. Jurkat T-cells were previously transduced with lentivirus containing pLVX-EF1a-IRES-zsGreen and sorted for green fluorescence, in order to obtain a zsGreen-expressing Jurkat cell line (Jurkat-IRES-zsGreen). Next, 1^×^10^6^ MT-2 were co-cultured with 1^×^10^6^ Jurkat-IRES-zsGreen for 1 h in the incubator (37 °C, 7% CO_2_). Cells were fixed and intracellularly stained (**se*e* flow cytometry).

### Flow cytometry

For analysis of cell viability, LIVE/DEAD Fixable Violet Dead Cell Stain Kit (Thermo Fisher Scientific) was used according to the manufacturer’s protocol, and cells were analyzed at the BD LSR II (BD Biosciences, Heidelberg, Germany).

For co-culture assays, cells were permeabilized using PBS supplemented with 0.5% FCS (FACS buffer) and 0.5% saponin, and stained with mouse anti-Gag-p19 (ZeptoMetrix, 0801082) or isotype control antibodies for 30 min at 4 °C. After two washing steps using FACS buffer supplemented with 0.3% saponin, cells were stained with secondary anti-mouse IgG (H + L) Alexa Fluor 647 (Thermo Fisher Scientific, A-21235) antibodies in FACS buffer supplemented with 0.5% saponin for 30 min at 4 °C. After two washing steps using FACS buffer supplemented with 0.3% saponin, cells were resuspended in PBS and analyzed at the AttuneNxT Flow Cytometer (Thermo Fisher Scientific). The FlowJo v10.8.1 software (BD Biosciences) was used for evaluation.

### Microscopy

For analysis of aggregate formation, MT-2 cells were treated with 1 µM Cav, CavOH or DMSO for 24 h. Next, cells were allowed to sit on µ-Slide 8 Well slides (Ibidi GmbH, Gräfelfing, Germany) slides at 37°C for 1 h, prior to analysis at the Nikon Eclipse TS2-FL inverted microscope equipped with a 20x objective. For every sample, at least 10 images per experiment were taken and analyzed using Fiji [67] considering at least 6900 cells and 180 aggregates per experimental condition.

For analysis of cellular conduits in Jurkat, cells were transfected with pME-p12WT-HA, pME-p12delta29-HA (pME-p8-HA), or pME by electroporation. At 6 h post transfection, 1 µM Cav, CavOH or DMSO was added. At 48 h post transfection, cells were adhered to µ-Slide 8 Well slides (Ibidi GmbH, Gräfelfing, Germany) at 37°C for 1 h. Next, cells were analyzed either with the Nikon Eclipse TS2-FL inverted microscope equipped with a 40x objective and the *TCapture software* (Version 5.1.1.0, Tucsen, Fuzhou, China), or with the Leica TCS SP5 confocal laser scanning microscope (Leica Microsystems GmbH, Wetzlar, Germany) equipped with a 63 × 1.4 HCX PL APO CS oil immersion objective lens and *LAS AF LiteSoftware* (Leica, Version 2.6.0). Conduits were counted manually, and at least ten visual fields per experimental condition in three independent experiments were analyzed considering at least 1400 cells per experimental condition.

### Protease inhibitor screening

Jurkat were treated with protease inhibitors from Protease Inhibitor Panel INHIB1 (Sigma-Aldrich) separately in varying concentrations for 4 h. Next, cells were electroporated with pME-p12WT and treated with the respective protease inhibitor for 24 h. Cells were lysed with the respective protease inhibitor added to corresponding lysis buffer, and proteins were separated by SDS-PAGE and analyzed via Western Blot. To analyze protease inhibitor toxicity, cells were stained with 4 µg/ml propidium iodide (PI) and analyzed at the BD LSR II (BD Biosciences).

### Statistics

Data were analyzed using GraphPad Prism 10. Normality of the dataset was tested using Shapiro-Wilk test. Non-normally distributed data were compared using Kruskal-Wallis test with Dunn’s multiple comparisons correction. p < 0.05 was considered to be significant, and statistics are denoted as *p < 0.05, **p < 0.01, ***p < 0.001, and ****p < 0.0001. Raw data and analyses are displayed in S1 Table.

## Supporting information

S1 Fig Commercial protease inhibitors do not interfere with p12 cleavage in Jurkat. **(A-H)** A commercial protease inhibitor panel was evaluated for its effects on p12 cleavage via immunoblot **(A-F)** and densitometric analysis **(G)**. Viability **(H)** was analyzed and normalized to respective solvent control. The mean of two independent experiments is depicted.
(TIF)

S2 Fig p12 is predicted to carry a signal peptide using PrediSi. The p12 wildtype (WT) sequence was analyzed with the signal peptide prediction tool PrediSi.
(TIF)

S3 Fig SEC11C is not downregulated upon transfection with siSEC11C in 293T. Representative immunoblot of p12 cleavage and SEC11C expression in 293T cells transfected with siRNA targeting SEC11C.
(TIF)

S4 Fig Hydrophobicity of *ORF-I* signal peptide like sequences is comparable between HTLV-1 genotypes. Calculation of the frequency of hydrophobic amino acids and the grand average of hydropathy (GRAVY) of the *ORF-I* signal peptide sequence of various HTLV-1 strains and genotypes. *n/a*: not available.
(TIF)

S5 Fig Gating strategy of Figure 3F. Dot plots of one representative repeat are shown.
(TIF)

S6 Fig Inhibition of p12 cleavage does not affect HTLV-1 cell-to-cell transmission from C91-PL to Jurkat. **(A-D)** Viral protein expression **(A)**, cell viability **(B)** and growth **(C)**, as well as virus release **(D)** of chronically HTLV-1 infected C91-PL cells treated with DMSO or increasing doses (0.2 µM, 1 µM or 2.5 µM) of either Cav or CavOH after 48 h of treatment. **(B-D)** Data shown as means ± SEM. n = 3. Statistical significance was determined using Kruskal-Wallis test. *p < 0.05, **p < 0.01.
(TIF)

S1 Table Data set used for analysis. Raw data and statistical analyses are listed for each individual subpanel of each Figure and Supporting Figure if applicable.
(XLSX)
