# Supplementary figures and images for: HTLV-1 p12 is cleaved to p8 by the signal peptidase complex and its inhibition impairs p8-dependent transmission
Source: PLoS Pathog. 2025 Oct 10;21(10):e1013570. doi: 10.1371/journal.ppat.1013570 (PMC12513584; doi:10.1371/journal.ppat.1013570)

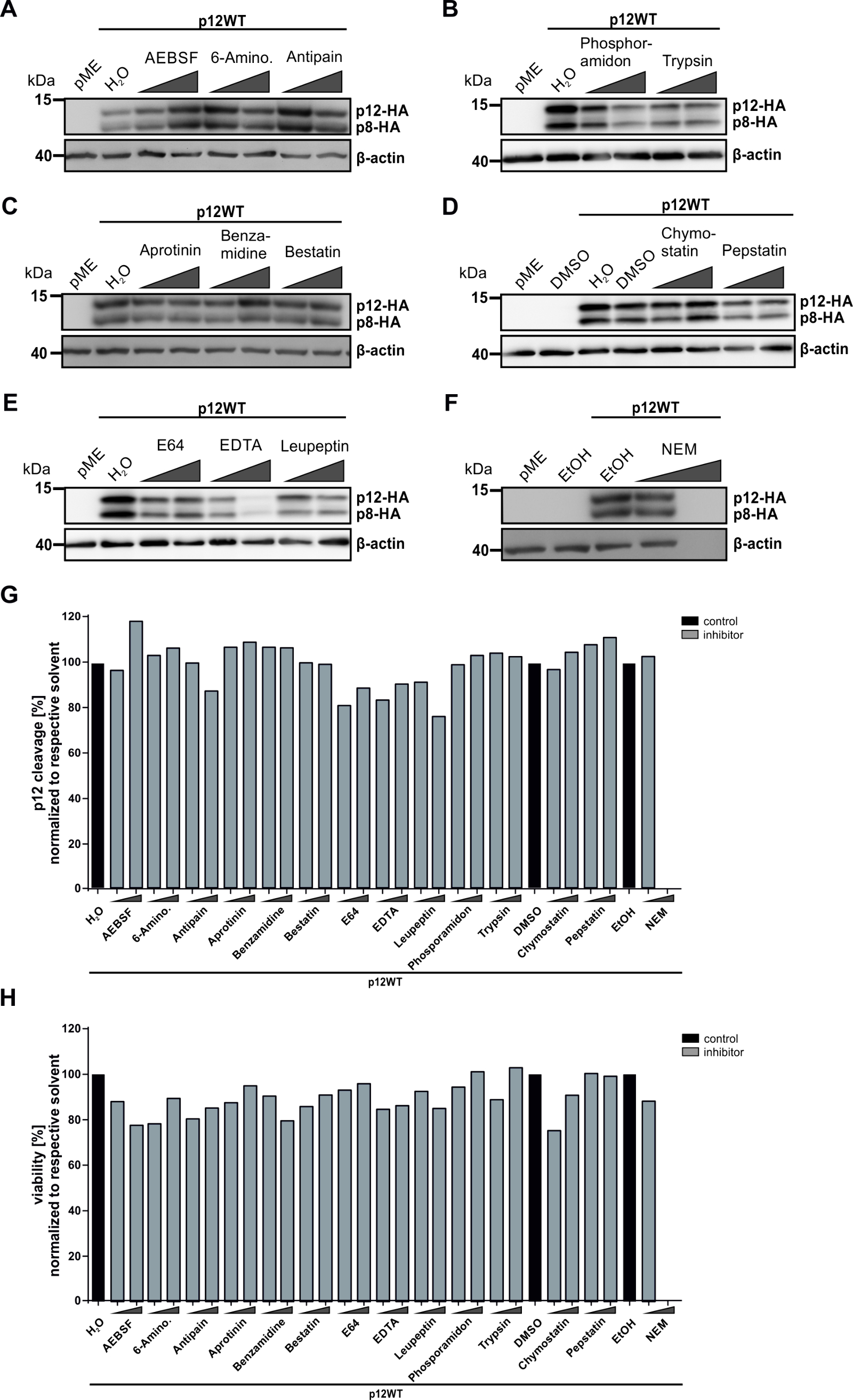

Supplement: S1 Fig — (A-H) A commercial protease inhibitor panel was evaluated for its effects on p12 cleavage via immunoblot (A-F) and densitometric analysis (G). Viability (H) was analyzed and normalized to respective solvent control. The mean of two independent experiments is depicted. (TIF) [file ppat.1013570.s001.tif]

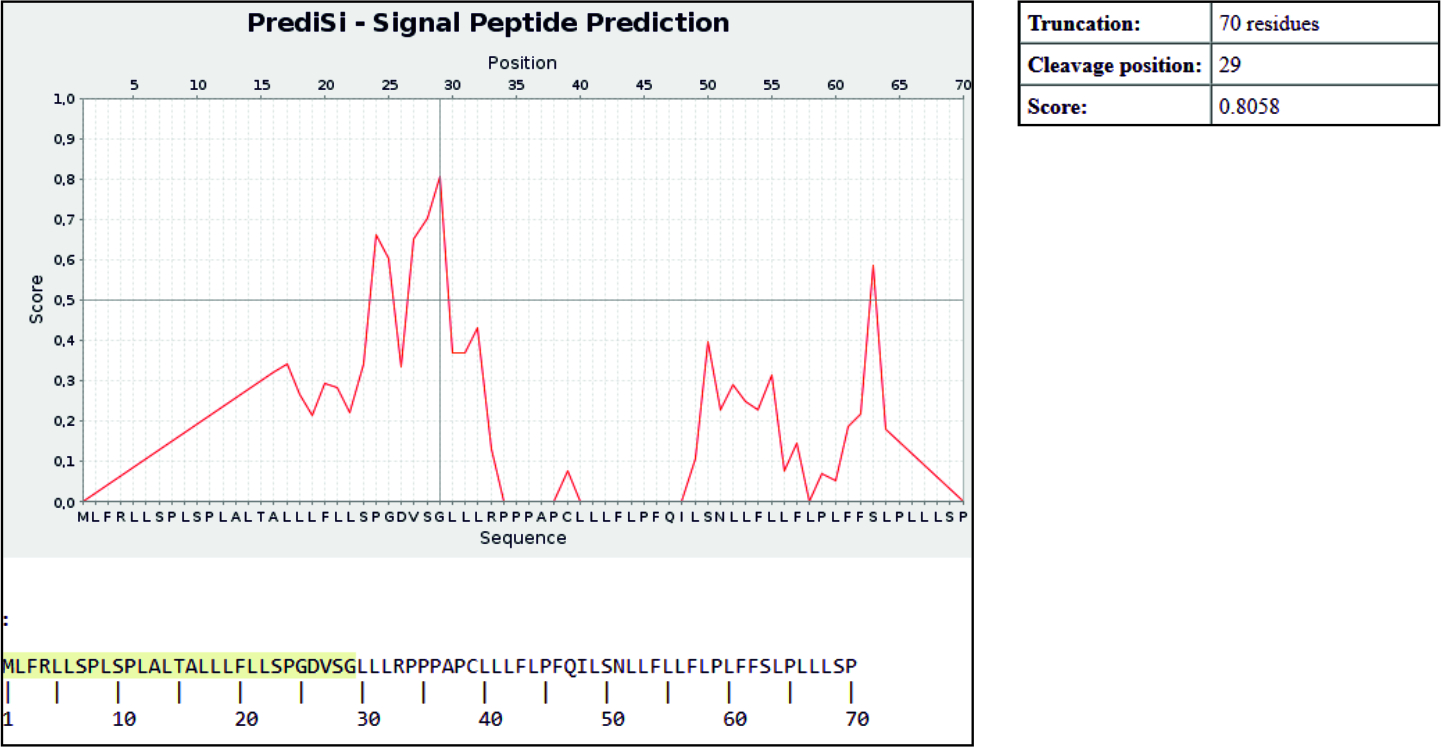

Supplement: S2 Fig — The p12 wildtype (WT) sequence was analyzed with the signal peptide prediction tool PrediSi. (TIF) [file ppat.1013570.s002.tif]

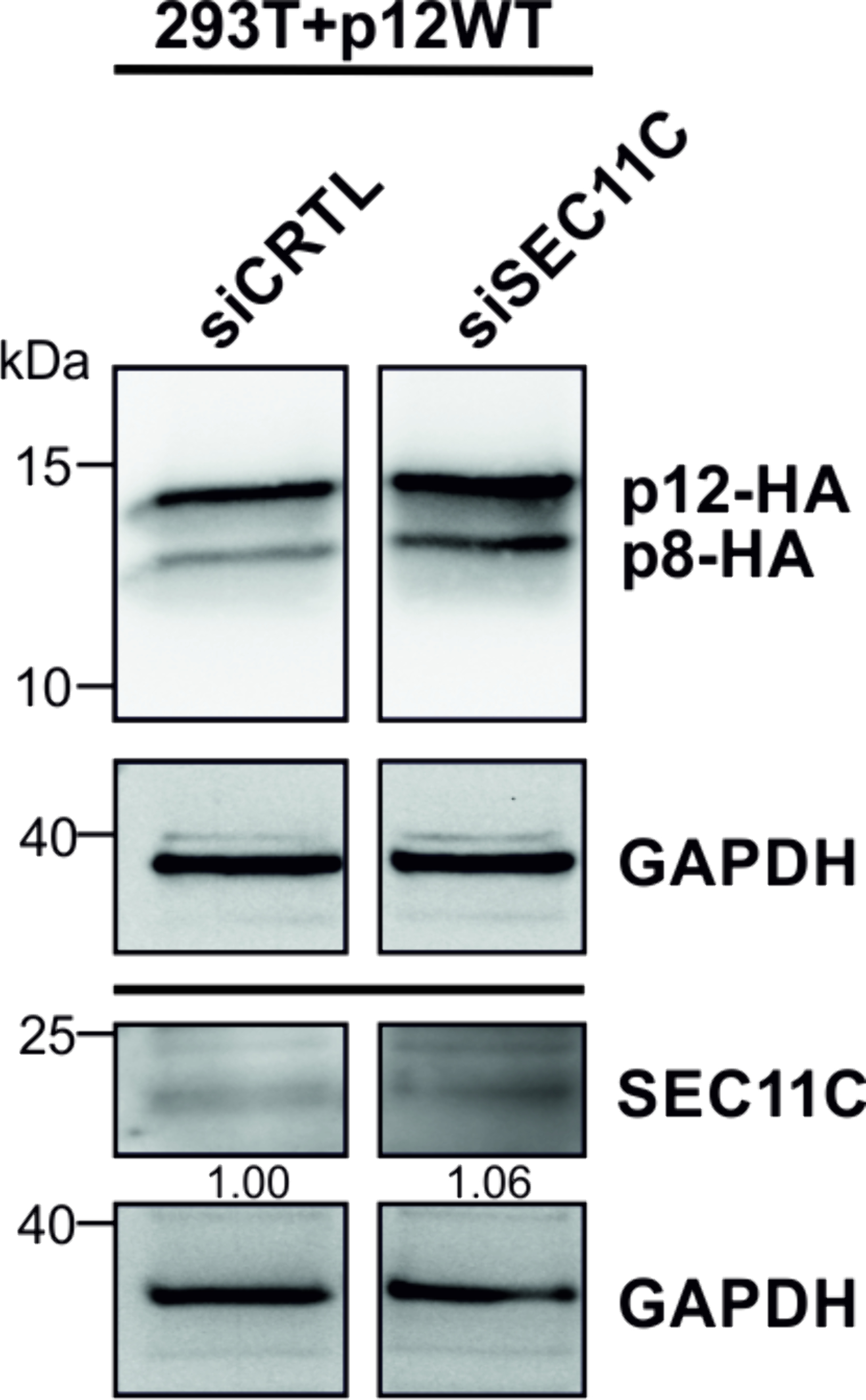

Supplement: S3 Fig — Representative immunoblot of p12 cleavage and SEC11C expression in 293T cells transfected with siRNA targeting SEC11C. (TIF) [file ppat.1013570.s003.tif]

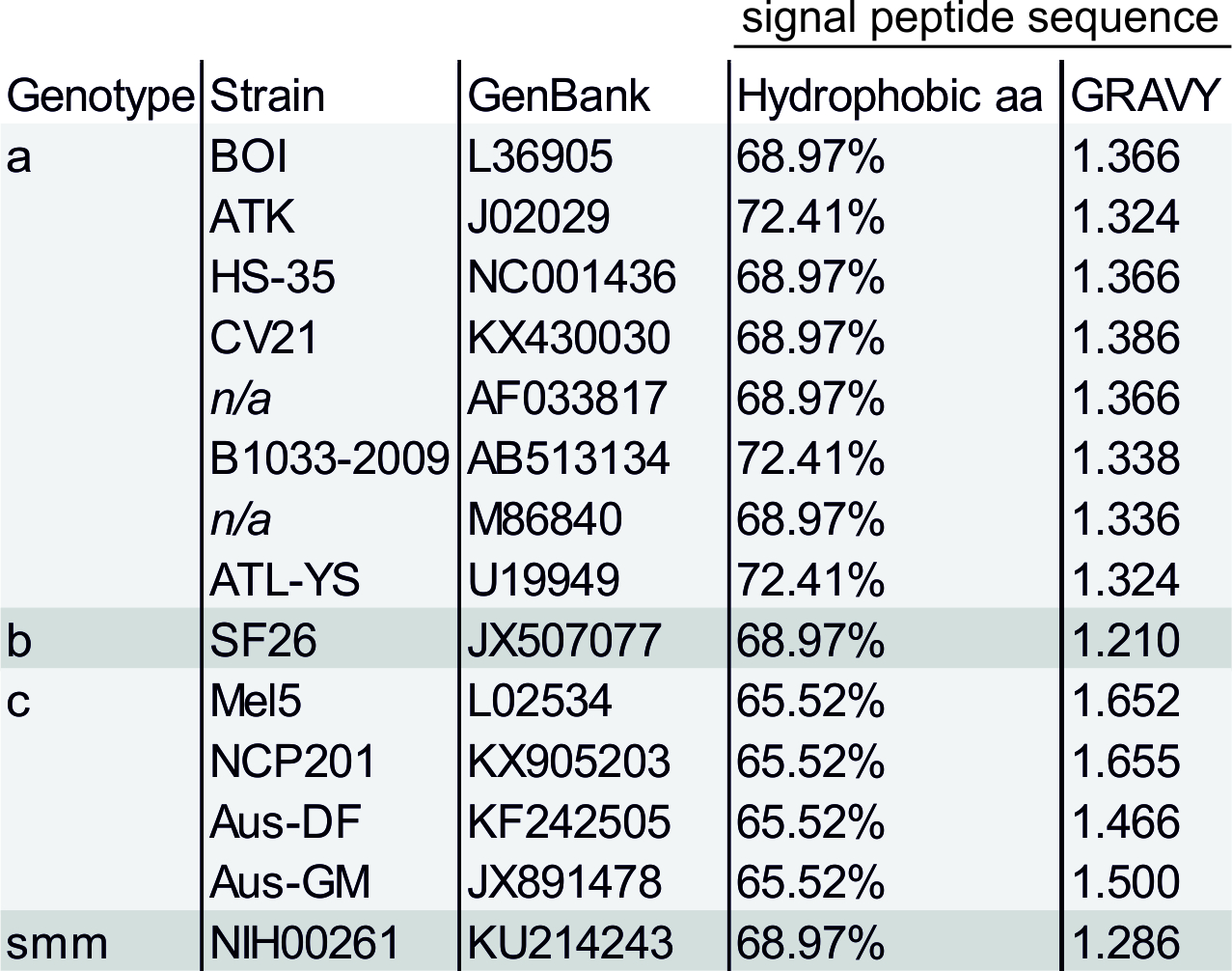

Supplement: S4 Fig — Calculation of the frequency of hydrophobic amino acids and the grand average of hydropathy (GRAVY) of the ORF-I signal peptide sequence of various HTLV-1 strains and genotypes. n/a: not available. (TIF) [file ppat.1013570.s004.tif]

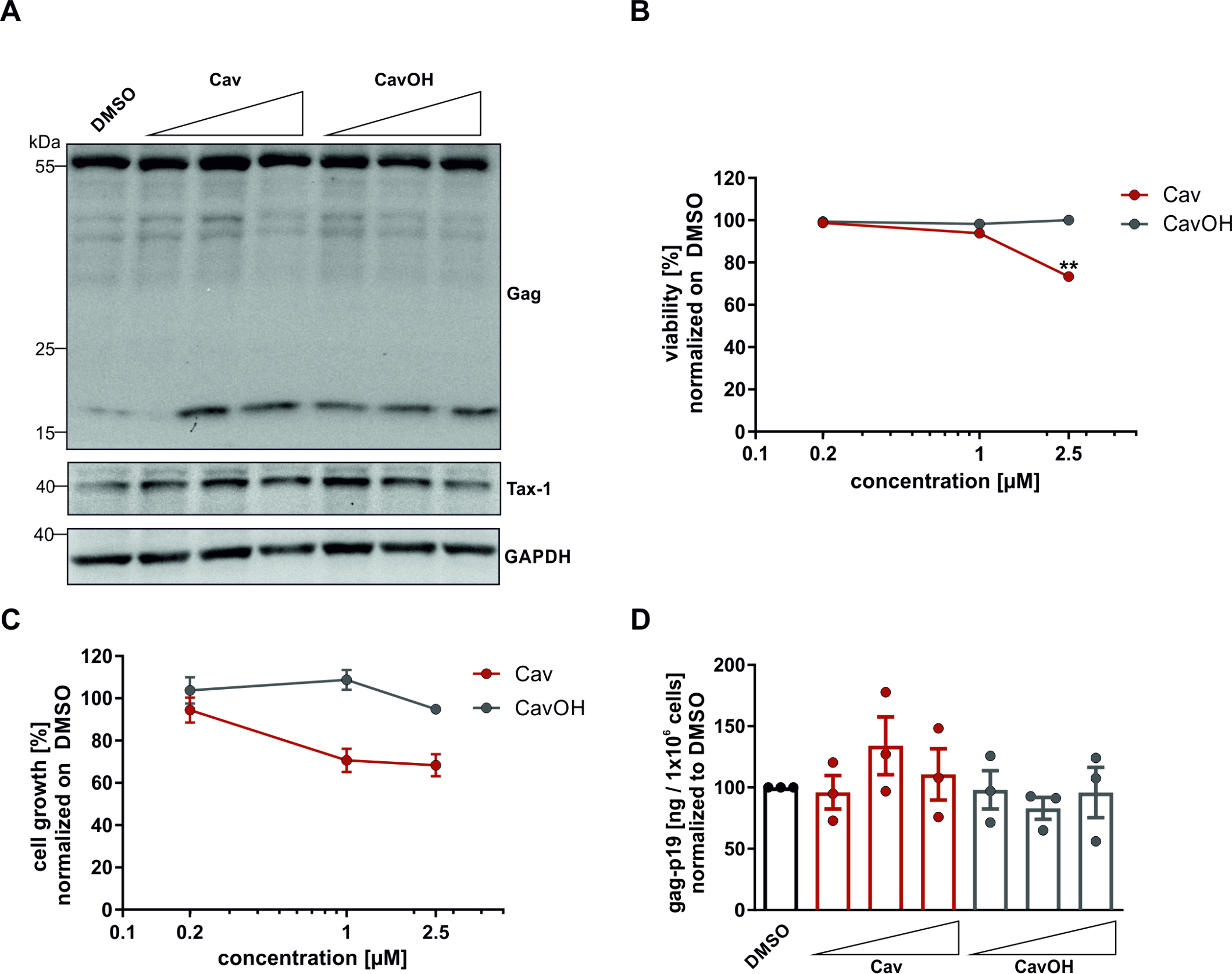

Supplement: S5 Fig — Dot plots of one representative repeat are shown. (TIF) [file ppat.1013570.s005.tif]

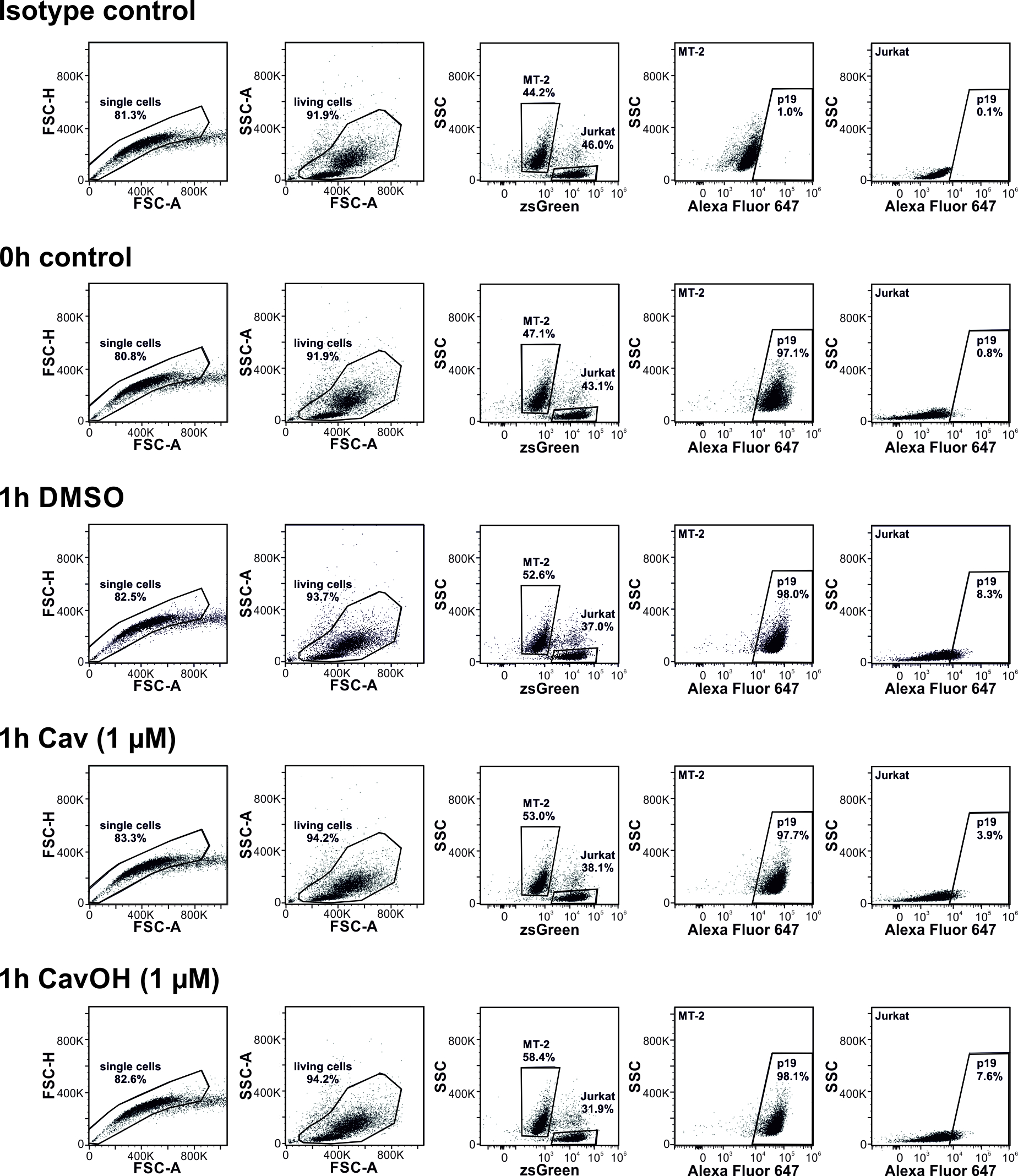

Supplement: S6 Fig — (A-D) Viral protein expression (A), cell viability (B) and growth (C), as well as virus release (D) of chronically HTLV-1 infected C91-PL cells treated with DMSO or increasing doses (0.2 µM, 1 µM or 2.5 µM) of either Cav or CavOH after 48 h of treatment. (B-D) Data shown as means ± SEM. n = 3. Statistical significance was determined using Kruskal-Wallis test. *p < 0.05, **p < 0.01. (TIF) [file ppat.1013570.s006.tif]
